# Neuromodulation of Dopamine D2 Receptors Alters Orbitofrontal Neuronal Activity and Reduces Risk-Prone Behavior in Male Rats with Inflammatory Pain

**DOI:** 10.1007/s12035-025-04781-0

**Published:** 2025-02-22

**Authors:** Margarida Dourado, Helder Cardoso-Cruz, Clara Monteiro, Vasco Galhardo

**Affiliations:** 1 Instituto de Investigação E Inovação Em Saúde (i3S), Pain Neurobiology Research Group, Universidade Do Porto, Rua Alfredo Allen 208, 4200-135 Porto, Portugal; 2 Instituto de Biologia Molecular E Celular (IBMC), Universidade Do Porto, Rua Alfredo Allen 208, 4200-135 Porto, Portugal; 3 Faculdade de Medicina (FMUP), Departamento de Biomedicina Unidade de Biologia Experimental (Floor4), Universidade Do Porto, Rua Doutor Plácido da Costa, 4200-450 Porto, Portugal; 4 Programa Doutoral Em Neurociências da FMUP, Universidade Do Porto, Rua Doutor Plácido da Costa, 4200-450 Porto, Portugal

**Keywords:** Orbitofrontal cortex, Dopamine D2 receptors, Inflammatory pain, Rodent gambling task, In vivo multielectrode extracellular recordings

## Abstract

Dopamine (DA) is believed to play a crucial role in maintaining the integrity of the rodent orbitofrontal cortex (OFC) networks during risk-based decision-making processes. Chronic pain conditions can lead to impaired DAergic signaling, which, in turn, may affect the motivational control of risk-based responses. Nevertheless, the neural mechanisms underlying this instability are poorly understood. In this study, we aimed to investigate whether this impairment is dependent on the activity of the DA D2 receptor (D2r). To address this hypothesis, we implanted bilateral matrices of multielectrodes into the OFC of male rats and recorded the neural activity while they performed a food-reinforced rodent gambling task (rGT). We evaluated behavioral performance and neural activity patterns before and after inducing a model of inflammatory pain – complete Freund’s adjuvant (CFA) model. Our findings revealed that rats treated with CFA exhibited an abnormal preference for the large/uncertain reward during rGT performance. This altered behavioral choice profile could be reversed by prior systemic administration of D2r ligands (0.05 mg/kg, quinpirole or raclopride), indicating a potential role of D2r in the decision-making process required for this task. The administration of these ligands at the specified dosages did not affect pain responses, but lead to a significant alteration of OFC neuronal activity that support goal-directed choice responses in the rGT. Finally, we found evidence that CFA-treated rats exhibit OFC functional changes, namely an upregulation of DA D1 receptor (D1r) and a downregulation of DA beta-hydroxylase (DH). These results demonstrate that the disruption of DAergic balance in the brain networks is crucial for the development of high-risk decision profiles during painful conditions.

**Supplementary Information:**

The online version contains supplementary material available at 10.1007/s12035-025-04781-0.

## Introduction

Emerging evidence suggests that decision-making under uncertainty, when outcome probabilities or magnitudes are unknown, involves complex processes primarily mediated by frontal lobes and mesocorticolimbic dopaminergic pathways [1, 2]. Within this framework, the orbitofrontal cortex (OFC) is essential for cost–benefit evaluations [3, 4], facilitating behavioral flexibility by continuously updating information for decision-making [5, 6]. Neuroimaging consistently shows OFC activation during reward-related and outcome monitoring tasks [7, 8], with OFC neurons selectively responding to reward-predictive stimuli [9, 10] and exhibiting sensivity to reward type and magnitude [11, 12].

Studies indicate that individuals with chronic pain show impaired reward processing and valuation task [13, 14]. Our prior research also found altered decision-making in pain-affected rodents, which favored uncertain, high-reward outcomes [15, 16]. Chronic pain, by overloading sensory processes, likely diminishes DAergic activity in the mesocorticolimbic pathway to the OFC [16–20], contributing to hypostimulation and decision-making impairments similar to those observed in neurological and psychiatric disorders [18, 21–23]. This hypoactivity can compromise cost–benefit control and impulsivity regulation over time [23–25]. The effects of pain extend beyond frontal areas, contributing to dopamine (DA) disturbances in other regions related to nociceptive processing. For instance, inflammatory pain in rodents decreases tonic DA levels while increasing D2 receptor (D2r) expression [26] and activating D2r can reduce pain-related behaviors [27]. Recent findings highlight that D2r in the prefrontal cortex not only supports risk aversion [28, 29], but also influences information processing across brain regions during cognitive tasks [30–33]. Collectively, this emphasizes D2r’s role in pain and cognitive-affective processing.

Despite these advances, it is unclear whether inflammatory pain affects DAergic function within the OFC and whether this disrupts risk-related information encoding essential for predict future outcomes on the basis of past and present experience. This study investigates whether D2r modulation can correct abnormal OFC neural representations of risk assessment in inflammatory pain. Through pharmacological intervention, we assessed the role of D2r in pain-related risk behavior during a rodent gambling task (rGT) and characterized the impact of inflammatory pain on DAergic markers in the OFC.

## Materials and Methods

### Animal Model and Ethical Statement

Experiments were conducted using adult male Sprague–Dawley rats (weight 275–325 g; Charles River Labs, Saint Germain, Neulles, France). Prior to surgery, the rats were housed in collective standard cages (type H, 3 per cage), which included environment enrichment. They were maintained in a 12-h light/dark cycle (lights on at 8:00 AM), with a constant-controlled temperature (22 ± 2ºC) and humidity (55 ± 5%). The training and recording sessions were consistently performed during the light phase, approximately at the same time each day. The testing room was moderately illuminated, sound attenuated, and featured in visual cues. All rats were food deprived to approximately 90–95% of their ad libitum feeding, while having unlimited access to water. Rats weights were checked daily after surgery, and their growth was monitored weekly using a standard curve for Sprague–Dawley rats as a reference. Prior to the commencement of any experimental protocol, the rats were habituated to handling by the experiments.

All procedures and experiments adhered to the guidelines set forth by the Committee for Research and Ethical Issues of the International Association for the Study of Pain [34], and the Ethical Guidelines for Animal Experimental of the European Community Directive (2010/63/CE). The experimental protocols received approval from the local Ethical Committee of Faculty of Medicine of University of Porto (Porto, Portugal) (approval ORBEA-81/2019 of 1–10–2019) and by national board of the Direção Geral de Alimentação e Veterinária (Lisbon, Portugal) (approval DGAV-28498–2019 of 01–12–2019). During the execution of the experimental protocols, at least one experimenter certified under category C by FELASA was present when animals were subjected to the procedures. Every effort was made to adhere to the 3R’s recommendations for animal experimentation, minimizing animal distress and using the minimum number of animals necessary.

### Stereotaxic Implantation of Multielectrodes for In Vivo Extracellular Neural Activity Recordings

Stereotaxic bilateral implantation of microelectrodes was performed following a previously described protocol in detail elsewhere [35]. Each multielectrode array was assembled in a 4 × 2 configuration, with a spacing of 250 µm between each row and 400 µm between columns [35]. For the placement of multielectrode arrays in the lateral OFC (LO), the following stereotaxic coordinates (in millimeters) relative to bregma were used to ensure accurate positioning: 3.7–4.7 mm anterior to bregma, ± 2.2–2.4 mm lateral to midline, and a depth of 4.5–5.5 mm [36]. After surgical procedures, an analgesic (ketoprofen, 5 mg/kg) and an antibiotic (enrofloxacin, 5 mg/kg) diluted 1/5 in saline solution (NaCl 0.9% w/v) were administered subcutaneously (s. c.) every 24 h for 5–7 days to improve post-operative recovery. Their overall health status was monitored daily.

### Inflammatory Pain Model

A persistent monoarthritis inflammatory pain model was induced by injecting 50 μl of complete Freund’s adjuvant (CFA; Sigma-Aldrich, Lisbon, Portugal) into the tibiotarsal joint of both hindpaws under brief isoflurane-induced anesthesia (henceforth referred CFA – experimental group). In a separate group for control purposes (henceforth referred sham—experimental group), rats were injected with an equivalent volume of saline solution. The assessment of the sensory threshold for noxious mechanical stimulation was conducted 1 h after each recording session using von Frey filaments (Somedic Inc., Sösdala, Sweden), following the previously described method [37]. Pressure values for sensory threshold response are presented in grams per square millimeter.

### Pharmacological Dopamine D2 Receptor Modulators

**Fig. 1:**
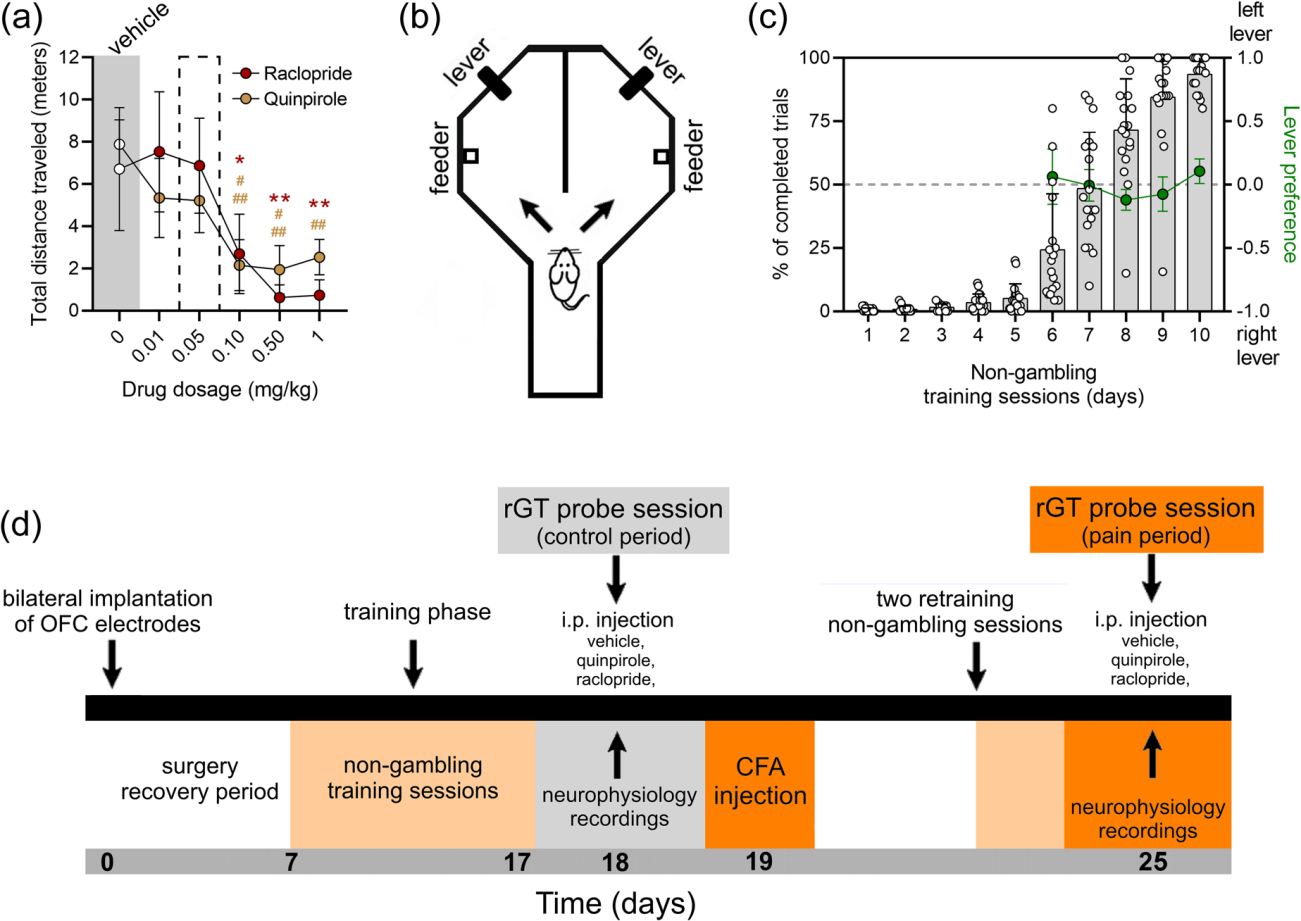
Rodent gambling task, training procedures, and experimental timeline. **a** Dose-dependent effect of systemic administration of dopamine D2r ligands on exploratory motor activity in a classical open-field arena. Quinpirole – D2/3r agonist (*n* = 6); Raclopride – D2r antagonist (*n* = 6). Control baseline (saline, 0.9% NaCl w/v). The selected dose of both D2r ligands applied during the rGT probe sessions is indicated by a black dotted rectangle. **b** Illustrative diagram of the rodent gambling task (rGT) arena used in this study. **c** Behavioral performance during the training phase under a non-gambling contingency showed a significant increase of the percentage of completed trials after 5 training sessions (left *y*-axis). The left/right lever balance of individual choice profiles during non-gambling sessions indicated no specific preferences for one side of the experimental arena (ratio of left/right choices measured by the choice preference index (right *y*-axis: green line calculated for the last 5 training sessions). **d** Timeline of experimental procedures. Briefly, each rat underwent bilateral implantation of a multielectrode array in the OFC. After the recovery period of 7 days, each rat started a 10-day rGT training protocol under a non-gambling training contingency. Following the training phase, each rat was tested in 2 rGT probe sessions under a gambling contingency. The first rGT probe session served as the control period, while the second probe session was performed 6 days after the injection of CFA (pain period). Values are presented as mean ± S.D. Statistical inference of the dose-dependent effect of pharmacological treatments, and side preference were performed using the non-parametric repeated-measures Friedman test followed by Dunn’s post-hoc test. * when *p* < 0.05 (raclopride), **/^##^ (quinpirole/raclopride) when *p* < 0.01, and.^###^ when *p* < 0.001 (quinpirole)

The DA D2/3r agonist quinpirole (QP) and the D2r antagonist raclopride (RC) were obtained from Sigma-Aldrich (Cat. No. 73625–62-4 and 98,185–20-7, respectively). These drugs were dissolved in aseptically prepared saline solution (NaCl 0.9% w/v). The drug doses were carefully selected to elicit maximal behavioral effects while preserving the necessary motor activity required for performing the rGT. To assess the dose-dependent effects on motor activity, an open-field arena (45 × 45 cm, with opaque walls measuring 40 cm high) was used. Six doses of QP and RC were tested: 0 (saline solution), 0.01, 0.05, 0.1, 0.5, and 1 mg/kg (Fig. 1a). During the rGT probe sessions, all rats received intraperitoneal (i. p.) injections of 1 ml/kg of either the vehicle (VH; NaCl 0.9% w/v), QP (0.05 mg/kg), or RC (0.05 mg/kg). Following the injection, rats were placed in a holding case for 20 min before the initiation of the rGT recording session.

### Rodent Gambling Task (rGT)

The rGT is based on an adapted version of the original protocol described in previous studies [15, 16, 38]. Briefly, the behavioral arena comprises a custom-built hexagonal arena with a distance of 45 cm between opposite walls. This arena is connected to a corridor measuring 10 cm in width and 30 cm in length (see Fig. 1b). The hexagonal arena is partially divided into two adjacent chambers by an opaque vertical wall measuring 30 × 30 cm. Each chamber contains a retractable lever and a food cup connected to a pellet dispenser (Coulbourn Instruments; Whitehall, PA, USA). Pellet dispensers and retractable levers are fully automated using the OpenControl software, which has been adapted for this specific task [39]. During the training phase, rats begin in the corridor and are trained to enter the central hexagonal hub. From there, they must choose between the left and right sides and press the exposed retractable lever to receive a reward (Cat. No. F0023; 45 mg chocolate-flavored sucrose pellet; Bioserv, Frenchtown, NJ, USA). After receiving the reward, the rat is removed from the testing arena and must wait 10 s before the start of the next trial. Each trial has a duration limit of 15 s and is classified according to the behavioral response: high-risk trial—rewarded (HR) or not rewarded (HNo); and low-risk trial—rewarded (LR) or not rewarded (LNo). If the rat reaches the trial duration limit without pressing any lever, an incomplete trial or omission is considered.

### Experimental Workflow

A graphical representation of the experimental timeline can be found in Fig. 1c. Following the recovery period from the surgical procedures (see details above), all rats underwent training to press the retractable levers using a non-gambling contingency. During this training phase, both retractable levers were programmed to a low-risk profile, dispensing one food-pellet every eight out of ten visits. This training phase lasted for 10 days, with each rat undergoing two daily sessions of 15 min each. To minimize potential biases, rats from different experimental groups were tested alternately. Only rats that achieved an 80% completion rate of lever presses during the non-gambling contingency training phase were selected to proceed with the testing protocol (Fig. 1d). The gambling phase consisted of two probe sessions, each comprising 90 trials. During these sessions, the levers were programmed with different probability of reward values. One lever was set to a non-gambling contingency (low-risk lever), dispensing one food pellet every nine out of ten visits, representing a small and certain reward profile. The other lever was set to a gambling contingency (high-risk lever), dispensing three food pellets every three out of ten visits, representing a larger and uncertain reward profile. This protocol was designed to achieve a balanced net gain for each lever in each testing session. The first rGT probe session (ahead referred as control period rGT session) took place 18 days after the implantation surgery. The second rGT probe session (ahead referred as pain period rGT session) was conducted 25 days after implantation surgery and six days after the peripheral injection of CFA. Two days prior to the second rGT probe session, all rats underwent retraining in the rGT using a non-gambling contingency (each session lasting 15 min in total).

### Extracellular Neuroelectrophysiological Recordings

During rGT performance, local neural activity in the OFC was recorded using two implanted multielectrode arrays, each with 8-channels, connected to a wireless headstage transmitter (W16; Triangle Biosystems – TBSI, Durham, USA). The analog signals from these electrodes were continuously transmitted to a Multineuron Acquisition Processor (MAP-16; Plexon Inc., Dallas, USA). To ensure accurate recording, the neural signals were pre-amplified (10,000–25000 times) and digitized at a sampling rate of 40 kHz. Single-unit waveforms were identified online by applying voltage–time threshold windows in SortClient 2.7 software (Plexon Inc., Dallas, TX, USA) and subsequently validated using a combination of automatic and manual sorting in Offline Sorter 2.8 software (Plexon Inc., Dallas, TX, USA). Sorting criteria adhered to previously established cumulative methods [40].

To collect additional data on the rat's position and movement within the rGT, an overhead video-tracking system (CinePlex 2.0; Plexon Inc., USA) provided synchronized video alongside the neural recordings, enabling precise extraction of behavioral event markers. The processed, sorted data were analyzed using NeuroExplorer software (NEX 4; Plexon Inc., USA), followed by further analysis in MATLAB (R2023a; MathWorks, USA).

### Quantification of OFC mRNA Levels of da Receptors and Associated Enzymes

Rats were deeply anesthetized with sodium pentobarbital (150 mg/kg, i. p.) and decapitated. Following decapitation, the brains were immediately removed, and specific brain regions, including the OFC, were dissected from both the left and right hemispheres. The dissected tissue was then immersed in RNALater (Ambion; Thermo Fisher Scientific) for preservation of RNA during subsequent processing and stored in a refrigerated condition. The brain was sectioned into thick coronal slabs (1 mm), and the area of interest was carefully dissected under surgical microscope. To minimize bias in the extent of the tissue collected, all samples were consistently obtained by the same experienced researcher. In this study, bilateral OFC samples were obtained from a total of 20 rats (control group, *n* = 10; and CFA group, *n* = 10). The CFA group samples were obtained 6 days after the injection of CFA. All tissue samples were rapidly frozen in liquid nitrogen and stored at −80ºC until further processing. For RNA isolation, the frozen tissue samples were mechanically disrupted, and total RNA was extracted using the TRIzol Plus RNA purification system (Life Technologies) following the manufacturer protocol. Subsequently, 1 µg of RNA was used for cDNA synthesis (ReverAid first Strand cDNA synthesis kit; Thermo Fisher Scientific). The PCR amplification was carried out using the StepOnePlus Real-Time PCR system (Applied Biosystems). SYBR Select Master Mix (Applied Biosystems) was used for the PCR reactions, and the results were analyzed using the StepOne software (Applied Biosystems). The real-time PCR reactions were performed in triplicates for each sample. The primer sequences used for amplifying specific target genes were as the follows: dopamine D1 receptor – D1r (accession number NM_012546.2), 5’-TGCTGCTGGCTCCCTTTC-3’ and 5’-GTTAATGCTCACCGTCTCTATGG-3’; dopamine D2 receptor—*D2r* (accession number NM_012547.1), 5'-TGGAGGTGGTGGGTGAG-3' and 5'-CAGCAGAGTGACGATGAAGG-3'; tyrosine hydroxylase—*TH* (accession number NM_012740.3) 5’CGTCCCCAAGGTTCATC-3’ and 5’-GGCTTCAAATGTCTCAAATACTT-3’; dopamine beta-hydroxylase—*DH* (accession number NM_013158.2), 5’-CACATCATCATGTATGAGGCC-3’and 5’-AGATTTCGGCAGTGCCATCT-3’; catechol-*O*-methyl-transferase—*COMT* (accession number NM_012531) 5′-GTGACGCGAAAGGCCAAATC-3′ and 5′-CAGGCCACATTTCTCCAGG-3′; monoamine oxidase—*MAO* (accession number NM_033653.1), 5’-CAGTATGGAAGGGTGATTCGCC-3’ and 5’-CAGACCAGGCACGGAAGG-3’; and glyceraldehyde 3-phosphate dehydrogenase—*GAPDH* (Accession number NM_017008.4), 5’-GCCATCAACGACCCCTTCAT-3’ and 5’-TTCACACCCATCACAAACAT-3’). The specificity of PCR reactions was confirmed using the melting curves of each gene. Amplifications were performed starting with a 2 min step for enzyme activation at 95ºC, followed by 2 min at 50ºC, and by 40 cycles at 95ºC for 30 s, 60ºC for 45 s, and 72ºC for 45 s. For each animal, the semi-quantitative expression of the gene of interest was performed according to the delta Ct method, using GAPDH as housekeeping gene.

### Anatomical and Histological Validation

After the final recording session, the rats underwent a terminal procedure. They were deeply anesthetized with sodium pentobarbital (150 mg/kg, i. p.) and transcardially perfused with 0.01 M phosphate buffer, pH 7.2, in saline solution (NaCl 0.9% w/v), followed by 4% (v/v) paraformaldehyde. The brains were carefully removed and post-fixed in 4% (v/v) paraformaldehyde for a duration of 4 h. To ensure proper preservation, the fixed brains were then stored in a 30% sucrose solution (w/v) before they were frozen and sectioned into 60 µm thick coronal slices. These brain slices were used to identify microscopically the precise locations of the implanted microelectrode arrays. The coordinates of the recorded regions were determined using the rat brain atlas [36]. Only rats with accurately positioned implanted arrays within the OFC region were included in the subsequent data analysis.

### Data Analysis and Representations

Several behavioral performance parameters were examined during rGT testing sessions. The rGT choice preference index was defined as the proportion of choices between both levers during the gambling probe sessions. It was computed for each block of 10 consecutive trials using the following formula: *(number of low-risk lever choices)—(number of high-risk lever choices) / (number of completed trials)* [38]. The choice preference index value oscillates between −1 and 1, where higher values (close ~ 1) indicate a strong preference for the low-risk lever (small/certain reward), whereas low values (close ~ −1) indicate a strong preference for the high-risk lever (large/uncertain reward). Additionally, we calculated the mean response latency to lever-press for each rGT probe session, as well as the percentage of omissions performed (or incomplete trials). To visually represent the temporal sequence of rGT responses on a trial-by-trial basis, we employed a color code to classify each trial. Specifically, we used “green/cyan” to represent low-risk trials, “red/orange” for high-risk trials, and “black” for omissions. Subsequently, this dataset was used to compute the rGT response curves based on lever preference (respective bottom panels). The response curves were smoothed using a mean activity window computed with 3-points per step (3 consecutive trials).

The activity of OFC neurons was analyzed for each rat by comparing the control and pain periods during rGT probe sessions. To characterize the temporal patterns of neuron firing during task performance, individual perievent time histograms (PETHs) were computed and plotted. The time-window for PETHs was set from 5 s before lever-press to 10 s after lever-press (lever-press response = 0 s). Each bin in the PETHs represented a 50-ms interval. Subsequently, this data was post-processed with a smooth Gaussian filter with a width of 3-points. The data was then exported to MatLab for additional analyses. The firing activity of each unit was transformed into Z-score (Z) values using the following equation: Z = (*x*—*m*) / *SD*, where *x* represents the raw firing rate of each neuron obtained from individual PETHs, and *m* and *SD* represent the mean and standard deviation of the neuron baseline activity, respectively.

To identify subgroups of neurons with similar activity patterns related to task behavioral responses, we employed an unsupervised hierarchical cluster analysis. First, the activity of each neuron was standardized by calculating the ratio between the average activity 5 s after lever-press and the average activity 5 s before lever-press (baseline). Neurons with firing rats below 0.5 Hz were excluded to minimize the impact of low-firing units. Hierarchical clustering of neuronal activity across the entire rGT probe session was then performed using the “clustergram” function in MatLab. We used Euclidean distance as the metric to assess similarity between pairs of neurons and applied the average linkage method for clustering. The resulting clusters were illustrated in a dendrogram, with colors assigned to highlight groups with similar activity patterns. The optimal cut point in the dendrogram was determined by selecting the point where the largest increase in distance (linkage less than a threshold of 3) occurred, ensuring distinct separation between clusters. Finally, the mean Z-score value for each cluster was computed to quantify the magnitude of the encoded neuronal choice responses.

### Statistical Analysis

All datasets were assessed for normality using the Kolmogorov–Smirnov test (with Dallal-Wilkinson Lilliefor corrected *p*-value) (GraphPad Prism 8.0; San Diego, CA, USA). The Wilcoxon matched-pairs signed rank test (*W*) or Mann–Whitney test (*MW*) were used to perform single comparisons, while the Kruskal–Wallis test (*KW*) (with post hoc Dunn´s test), Friedman test (*Fd*) (with post hoc Dunn’s test) or two-factors ANOVA (with post hoc Bonferroni test or Tukey’s test) to perform multiple comparisons. Statistically significant effects were considered when *p*-values were below an *α*-threshold of 0.05. All independence tests were two-tailed. Results are expressed as mean ± standard deviation (S. D.) or as mean ± standard error of the mean (S.E.M.).

## Results

### Dose-dependent Effects of D2r Ligands on Motor Activity

Given that systemic administration of D2r modulators is known to induce motor dysfunction in rodents, potentially affecting behavioral performance on the rGT [41, 42], we selected the highest dosage of each pharmacological modulator (0.05 mg/kg for both) that did not induce motor alterations during the test in a classical open-field arena (OP) (Fig. 1a). Data obtained from the OP arena revealed a significant dose-dependent effect of both drugs on motor activity (quinpirole: *Fd* = 24.86, *p* < 0.0001; and raclopride: *Fd* = 25.48, *p* < 0.0001; Fig. 1a). Post hoc analysis for both D2r ligands indicated a significant decrease in motor activity compared to baseline (saline administration) at the higher doses (quinpirole: 0.10 and 0.50 mg/kg *p* < 0.001, and 1 mg/kg *p* < 0.01; raclopride: 0.10 mg/kg *p* < 0.05, 0.50 and 1 mg/kg *p* < 0.01, Dunn’s post hoc test, Fig. 1a).

### rGT Performance During Learning Phase

A total of 18 male Sprague–Dawley rats were included in the experiments, with 6 rats assigned to each D2r ligand and vehicle control group. All rats included in this study achieved a minimum of 80% lever presses during the last learning training session under the non-gambling protocol (Fig. 1c). None of the rats displayed laterality towards a specific lever or rGT arena side during the learning phase (last 5 sessions: *Fd* = 4.30, *p* = 0.3665; Fig. 1c). Following the learning phase, behavioral and neuroelectrophysiological measurements were conducted in two periods: before and after the establishment of an inflammatory chronic pain model (see timeline, Fig. 1d).

### Pain-related rGT Performance During Dopamine D2r Ligands Neuromodulation

**Fig. 2:**
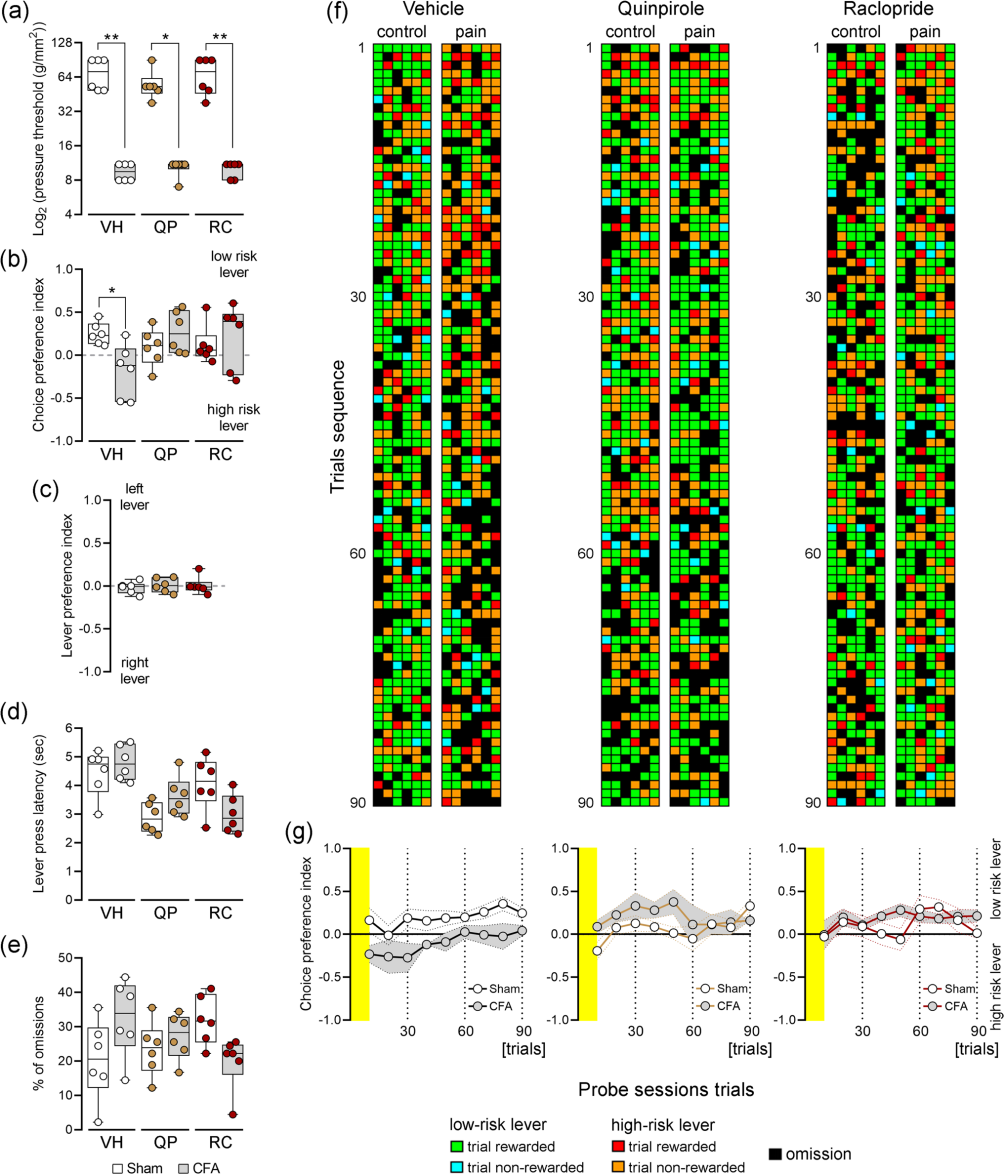
Pain-related rGT performance during modulation of dopamine D2r.** a** Systemic administration of D2r pharmacological modulators does not alter the threshold for pain responses to mechanical stimulation (von Frey filaments test). **b** Choice preference index during rGT probe sessions. **c** Lever preference measured in non-gambling retraining sessions conducted before the second rGT probe session (pain period). **d** Response latency to lever press during rGT probe sessions. **e** Percentage of omissions (incomplete trials) during rGT probe sessions. **f** Trial-by-trial response maps during rGT control and pain probe sessions. Each column of each panel represents the response of a rat during an entire rGT probe session. **g** Comparisons between rGT preference response activity curves. The yellow area on the panels indicates the first 10 trials of the probe session (vehicle administration: left panel, quinpirole administration: central panel, and raclopride administration: right panel). Values are presented as mean ± S.D. Comparisons between the experimental groups and pharmacological treatments were performed using the non-parametric Kruskal–Wallis test followed by Dunn’s post-hoc test. *when *p* < 0.05, and **when *p* < 0.01. VH, vehicle (VH, *n* = 6 rats); quinpirole (QP, *n* = 6 rats); and raclopride (RC, *n* = 6 rats). Supplementary material is provided in Figure S1 (Appendix A – Supplementary data)

First, we assessed the impact of systemic administration of D2r ligands on the mechanical threshold for noxious stimulation using the von Frey test (Fig. 2a). Our results demonstrated that all rats developed mechanical allodynia 6 days after CFA injection (*KW* = 28.03, *p* < 0.0001). Furthermore, systemic administration of D2r ligands at the selected concentration (0.05 mg/kg for both) did not revealed antinociceptive effects (sham period vs. CFA period: vehicle treatment, *p* < 0.01; quinpirole treatment, *p* < 0.05; and raclopride treatment, *p* < 0.01; post hoc Dunn’s test). The choice preference index of the rGT probe sessions exhibited characteristic patterns (Fig. 2b). Consistent with our previous findings [15], rats treated with the vehicle before CFA injection displayed a clear tendency to select the small and certain reward (low-risk lever). However, their behavior shifted towards a large and uncertain reward profile after CFA injection (high-risk lever). Importantly, rats treated with quinpirole or raclopride showed a clear risk-averse profile during both the control and pain periods. Statistical analysis demonstrated a significant effect between experimental groups and pharmacological treatments (*KW* = 12.66, *p* = 0.0410); moreover, p*ost hoc* analysis showed that rats treated with vehicle exhibited a low-risk preference during the control rGT session and an opposite preference during the pain period rGT probe session (*p* < 0.05; post hoc Dunn’s test, Fig. 2b). During the period of both rGT probe sessions, and immediately preceding the pain period probe session, each rat underwent testing in two re-training non-gambling sessions (Fig. 2c). Analysis of left and right lever choice revealed no specific preferences for either side of the rGT arena (ratio of left/right choices measured by the choice preference index; *KW* = 0.18, *p* = 0.9201). These data suggest that repeated exposure to the behavioral arena did not influence the preference signature of these rats between sessions. Another interesting point to consider is the response latency to lever press and the occurrence of omissions during the rGT probe sessions, as these factors can potentially influence behavioral performance. The response latencies to the lever press are depicted in Fig. 2d. Experimental data analysis revealed a significant effect in response latency to lever press (*KW* = 18.88, *p* = 0.0020), indicating different response regimes among the tested animals. However, post hoc test did not reveal any significant differences between control and pain periods, suggesting that the response latency was not significantly affected by the pain induction. Regarding the percentage of omissions during rGT probe sessions (Fig. 2e), no significant differences were observed among the experimental groups (*KW* = 10.17, *p* = 0.0707) indicating a similar rate of omissions across different treatment conditions. To provide a comprehensive overview of the preference response profile in the rGT, we utilized a color code to classify each type of trial performed. Figure 2f illustrates this response map for each complete probe session, considering the specific pharmacological treatment and control and pain periods. Additionally, for each pharmacological treatment, we calculated the average choice preference index throughout the entire rGT session (Fig. 2g). For rats treated with vehicle, it is evidence that after CFA injection, they shifted their preference towards the large and uncertain rewards compared to the control period. However, it is important to note that this profile is more prominent in the initial phase of rGT probe session (Fig. 2g, left panel). Conversely, rats treated with quinpirole or raclopride after CFA injection exhibited a reinforced preference for the small and certain rewards (Fig. 2g, middle and right panels).

### Pain-related OFC Activity During rGT Performance

**Fig. 3:**
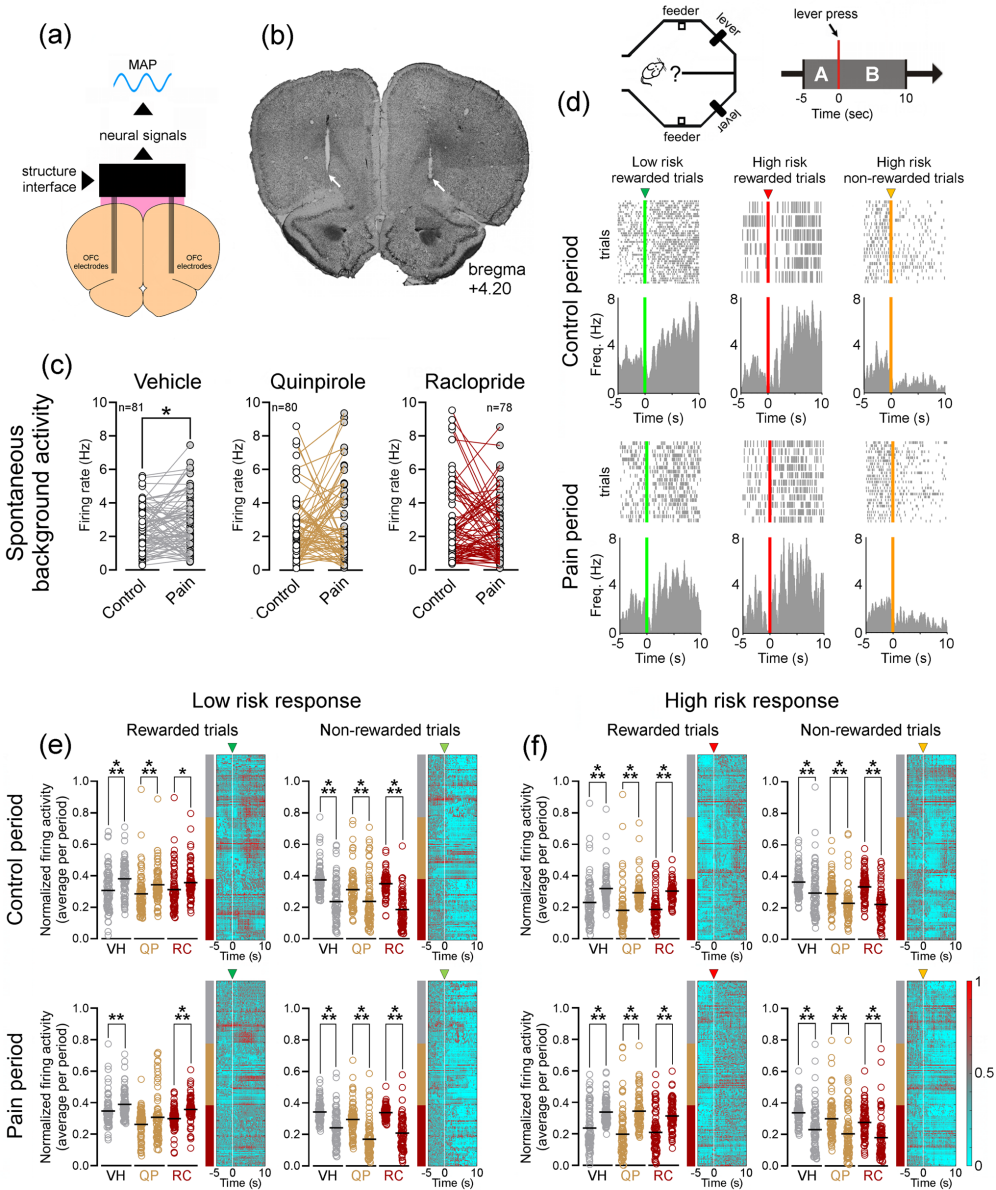
Experimental recording setup, tissue validation, and OFC neuronal task-related activity. **a** Experimental setup used for neuronal signals acquisition in the OFC. **b** Coronal microphotograph illustrating the bilateral implanted microelectrodes structure track. The recording locations are indicated by the white arrows. **c** Spontaneous mean background activity of the recorded units during the control and pain periods on an open-field arena. Units recorded in the groups of rats treated with vehicle revealed a significant increase in firing activity after CFA injection. **d** Perievent time histograms (PETHs) of OFC neurons recorded during low- and high-risk rewarded trials (left and central panels, respectively) and high-risk non-rewarded trials (right panel) in the rGT. **e** Dot plots on the left illustrate the comparison of normalized mean firing activity of recorded units before and after rGT lever press during low-risk rewarded and non-rewarded trials (left and right columns for each treatment respectively). The right heatmaps represent the oscillatory activity during the activity interval considered. **f** Similar to (**e**), but for high-risk responses. Top panels represent the mean activity during control period rGT session, while bottom panels represent the mean activity during pain period rGT session. VH, vehicle treatment; QP, quinpirole treatment; and RC, raclopride treatment. Non-parametric Wilcoxon match-pairs signed rank test was used to compare spontaneous activity between experimental periods, while non-parametric Kruskal–Wallis test followed by Dunn’s post hoc-hoc test was used to compare lever press responses between experimental groups and pharmacological treatments. * when *p* < 0.05, ** when *p* < 0.01, and *** when *p* < 0.001. Supplementary material is provided in Figures S1, S2, and Table S1 (Appendix A – Supplementary data)

To study the OFC activity during the rGT, we implanted two multielectrode arrays bilaterally in the OFC per rat. Figure 3a illustrates the experimental setup used to record neural activity in the OFC. The correct targeting of multielectrode array implantation in the OFC was verified after behavioral studies through brain cryosectioning. In Fig. 3b, we provide an example of the bilateral multielectrode structure track and tissue recording positions (indicated by white arrows). We obtained extracellular single-unit neural recordings from 18 rats in 6 rGT probe sessions. A total of 239 single-units were recorded (13.28 ± 0.19 neurons per rat): 81 units from vehicle-treated rats, 80 units from quinpirole-treated rats, and 78 units from raclopride-treated rats (for detailed information on the classification of recorded units based on their firing properties and waveform characteristics, please refer to Appendix A—Supplementary data – Table S1). To test the impact of pharmacological treatments, we compared the spontaneous background activity (during 10 min) before and after CFA injection (6 days) on an open-field arena (Fig. 3c). We observed a significant increase in OFC firing activity during the pain period for vehicle-treated rats (*p* = 0.0407; Fig. 3c, left panel). However, for quinpirole- and raclopride-treated rats, we found no significant changes in background activity (*p* = 0.7438, and *p* = 0.3453; Fig. 3c, central and right panels).

Next, to examine whether the recorded neurons exhibited task-related activity, we analyzed the neuronal activity during lever press using PETHs. Figure 3d presents an example of the activity of a neuron recorded during the control and pain periods across low- and high-risk rewarded trials, as well as high-risk non-rewarded trials. Due to the limited number of low-risk non-rewarded trials in each rGT session, we chose to exclude the representation of the corresponding PETHs. As shown in Fig. 3e and 3f, during low- and high-risk rewarded trials, the majority of the recorded neurons exhibited an increase in firing activity following the lever press. In contrast, the majority of the recorded neurons decreased their activity during non-rewarded trials. In the case of low-risk responses (Fig. 3e), rewarded trials were accompanied by an increase of firing activity (control period: KW = 49.65, *p* < 0.0001, post hoc Dunn’s test vehicle and quinpirole *p* < 0.001 and raclopride *p* < 0.05 (Fig. 3e, top left panel); and pain period: KW = 105.40, *p* < 0.0001, post hoc Dunn’s test vehicle *p* < 0.01 and raclopride *p* < 0.001 (Fig. 3e, bottom left panel)). However, no significant differences were observed for quinpirole-treated rats during the pain period. In the case of low-risk non-rewarded trials, after the lever press, the majority of neurons decreased their activity (control period: KW = 149.50, *p* < 0.0001, post hoc Dunn’s test all *p* < 0.001 (Fig. 3e, top right panel); and pain period: KW = 183.20, *p* < 0.0001, post hoc Dunn’s all *p* < 0.001 (Fig. 3e, bottom right panel)). Similar to low-risk responses, high-risk rewarded responses were characterized by an increase in OFC activity (control period: KW = 149.80, *p* < 0.0001, post hoc Dunn’s test all *p* < 0.001 (Fig. 3f, top left panel); and pain period: KW = 122.10, *p* < 0.0001, post hoc Dunn’s test all *p* < 0.001 (Fig. 3f, bottom left panel)), and non-rewarded trials by a decrease in activity after lever press (control period: KW = 115.60, *p* < 0.0001, post hoc Dunn’s test all *p* < 0.001 (Fig. 3f, top right panel); and pain period: KW = 109.50, *p* < 0.0001, post hoc Dunn’s test all *p* < 0.001 (Fig. 3f, bottom right panel)). These findings collectively suggest that the reward outcome plays a significant role in shaping firing patterns in the OFC.

### Impact of D2r Modulation on the OFC Neural Flexibility During rGT

**Fig. 4:**
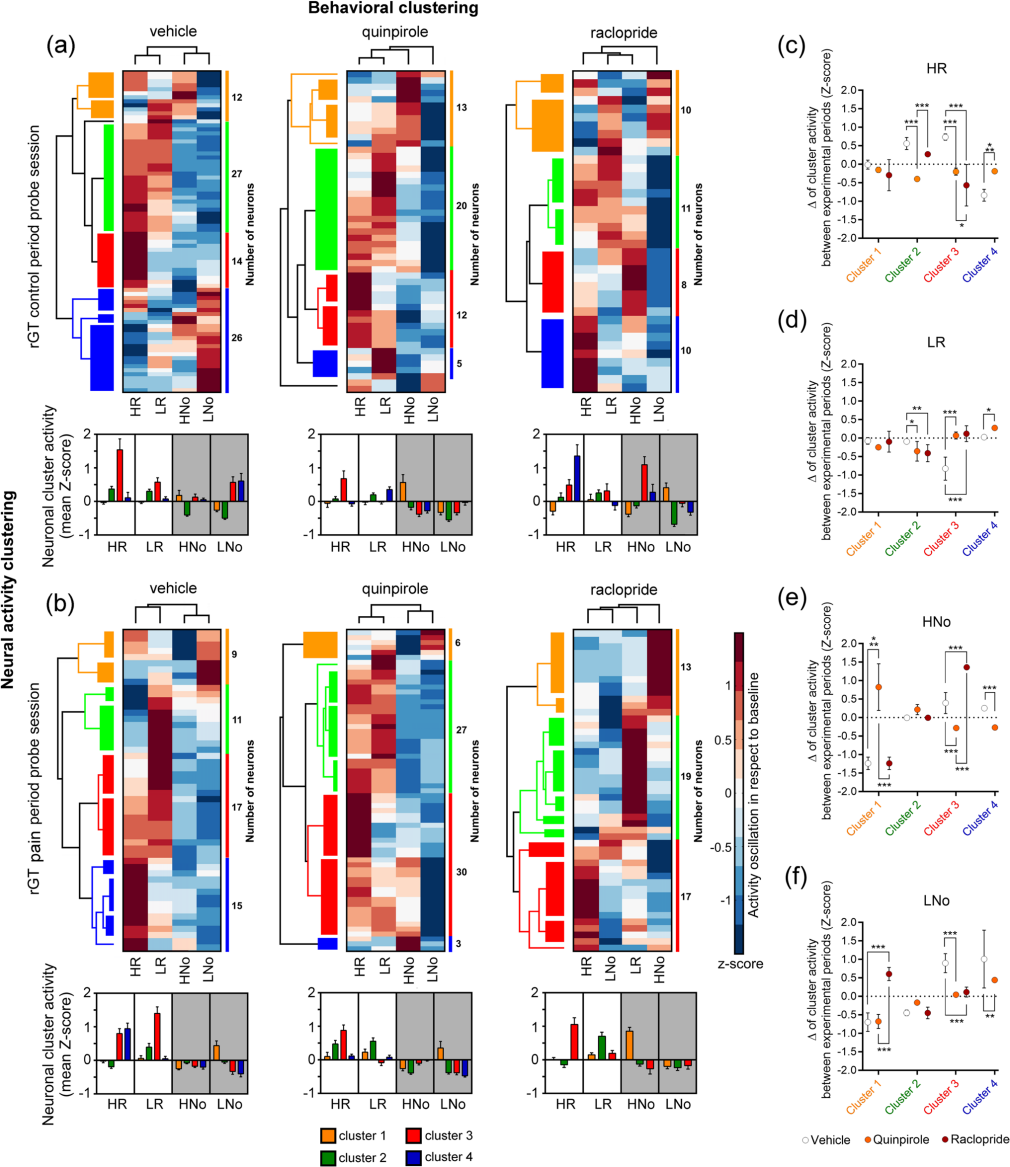
Blockade of dopamine D2r reduces the flexibility of OFC neuronal clusters to encode multiple rGT responses in CFA-treated rats. Graphical representations of two-dimensional unsupervised hierarchical clustering of neural activity profiles in respect to behavioral responses during the (**a**) control period, and (**b**) pain period rGT probe sessions. In the top panels, the activity of each unit is represented in rows, while the behavioral responses are represented in columns. Firing activity is depicted using a color scale, where warm colors (red) indicate an increase in firing compared to the baseline (before lever press), and cold colors (blue) indicate a decrease in firing. The values in the right column of each panel indicate the number of neurons included in each cluster. In left dendrograms of each panel, the height of each group of colored branches represents the similarity between units’ activity. Each bottom panel corresponds to the averaged activity of each cluster of units (mean Z-score) per rGT response. Values are presented as mean ± SEM. Variation of activity for each neural cluster between the control period and the period following CFA injection: **c** high-risk rewarded trials, **d** low-risk rewarded trials, **e** high-risk non-rewarded trials, and (**f**) low-risk non-rewarded trials. Values are presented as mean ± S.D. Low-risk trials: LR (rewarded) and LNo (non-rewarded); and High-risk trials: HR (rewarded) and HNo (non-rewarded)

Considering the variability of individual neuronal responses during rGT performance, the subsequent step involved employing unsupervised hierarchical cluster analysis to identify and organize the response signatures based on their activity profiles (Fig. 4). To ensure accurate results, recorded units with firing activity below 1 Hz were excluded, as they provided a limited number of spikes. The units not considered in this analysis were as follows: vehicle-treated rats (control period: *n* = 2, and pain period: *n* = 29 units), quinpirole-treated rats (control period: *n* = 30, and pain period: *n* = 14 units), and raclopride-treated rats (control period: *n* = 39, and pain period: *n* = 29 units). The resulting dendrograms (cluster trees) from the hierarchical cluster analysis were organized into color-coded groups. The height of each group of colored branches represented the similarity between the neuronal activity responses of the recorded units. Qualitative analysis revealed variations in the magnitude of response specialization between the control (Fig. 4a, top panels) and pain periods (Fig. 4b, top panels). The computed clusters demonstrated that the majority of recorded units exhibited preferential responses to rewarded trials. Another interesting observation was the flexibility or the ability of each cluster of neurons to encode more than one behavioral response in the rGT. This phenomenon was particularly evident during the rGT control period sessions (Fig. 4a), whereas during the pain period each cluster of neurons displayed reduced flexibility in encoding different responses (Fig. 4b). In fact, this specialization of neurophysiological signatures was especially pronounced in raclopride-treated rats during the pain period (Fig. 4b, bottom right panel).

Next, we analyzed the variation in activity for each neural cluster between the control period and the period following CFA injection. For high-risk rewarded trials (Fig. 4c), analysis of variance revealed a significant effect of experimental groups (two-way ANOVA: F_(2,60)_ = 16.15, *p* < 0.0001) and OFC neuronal clusters (F_(3,60)_ = 16.02, *p* < 0.0001). Moreover, post hoc analysis (Tukey’s test for multiple comparisons) indicated significant effects in cluster 2 (vehicle vs. quinpirole: *p* < 0.001; and quinpirole vs. raclopride: *p* < 0.001), cluster 3 (vehicle vs. quinpirole; *p* < 0.001; vehicle vs. raclopride: *p* < 0.001; and quinpirole vs. raclopride: *p* < 0.05), and cluster 4 (vehicle vs. quinpirole: *p* < 0.001). For low-risk rewarded trials (Fig. 4d), analysis of variance revealed a significant effect of experimental groups (F_(2,60)_ = 6.95, *p* = 0.0019) and OFC neuronal clusters (F_(3,60)_ = 16.25, *p* < 0.0001). Moreover, post hoc test indicated significant effects in cluster 2 (vehicle vs. quinpirole: *p* < 0.05; and vehicle vs. raclopride: *p* < 0.01), and cluster 3 (vehicle vs. quinpirole: *p* < 0.001; and vehicle vs. raclopride: *p* < 0.001), and cluster 4 (vehicle vs. quinpirole: *p* < 0.05). For high-risk non-rewarded trials (Fig. 4e), analysis of variance revealed a significant effect of experimental groups (F_(2,60)_ = 9.72, *p* = 0.0002) and OFC neuronal clusters (F_(3,60)_ = 70.19, *p* < 0.0001). Moreover, post hoc test indicated significant effects in cluster 1 (vehicle vs. quinpirole: *p* < 0.001; and quinpirole vs. raclopride: *p* < 0.001), cluster 3 (vehicle vs. quinpirole: *p* < 0.001; vehicle vs. raclopride: *p* < 0.001; and quinpirole vs. raclopride: *p* < 0.001), and cluster 4 (vehicle vs. quinpirole: *p* < 0.001). For low-risk non-rewarded trials (Fig. 4f), analysis of variance revealed a significant effect of experimental groups (F_(2,60)_ = 6.63, *p* = 0.0025) and OFC neuronal clusters (F_(3,60)_ = 45.23, *p* < 0.0001). Moreover, post hoc test indicated significant effects in cluster 1 (vehicle vs. raclopride: *p* < 0.001; and quinpirole vs. raclopride: *p* < 0.001), cluster 3 (vehicle vs. quinpirole: *p* < 0.001; and vehicle vs. raclopride: *p* < 0.001), and cluster 4 (vehicle vs. quinpirole:* p* < 0.01).

### mRNA Expression Levels of OFC da Receptors and Related Enzymes

**Fig. 5:**
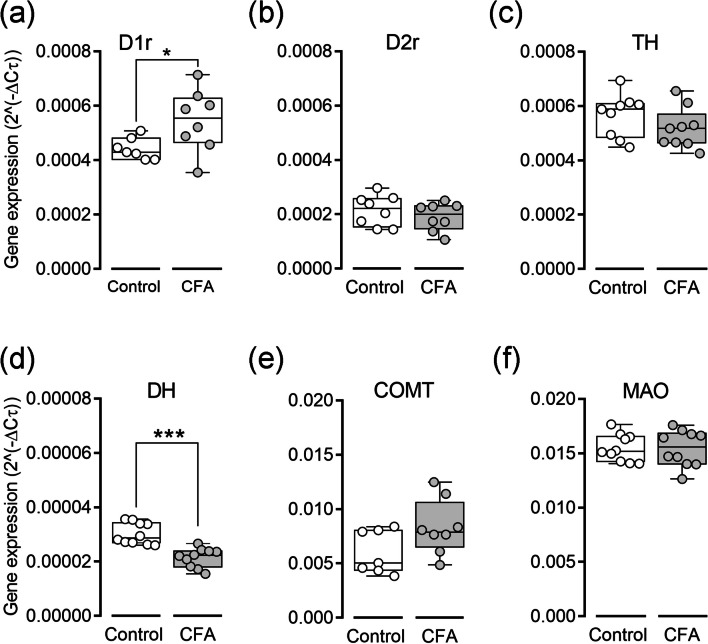
Inflammatory pain did not result in significant changes in the local expression of DA receptors and related enzymes in the OFC. Gene expression was analyzed using real-time PCR with GAPDH serving as the housekeeping gene. Each bar represents the average of individual tissue samples that were run separately (control and CFA groups). **a** Dopamine D1 receptor, D1r. **b** Dopamine D2 receptor, D2r. **c** Tyrosine hydroxylase, TH. **d** Dopamine beta-hydroxylase, DH. **e** Catechol-*O*-methyl-transferase, COMT. **f** Monoamine oxidase, MAO. Comparisons between the experimental groups were conducted using the non-parametric Mann–Whitney test for unpaired samples. Values are presented as mean ± S. D. * when *p* < 0.05, and *** when *p* < 0.001

Finally, we assessed whether inflammatory pain could induce changes in the expression of DA receptors and related enzymes in the OFC (Fig. 5). This analysis was conducted in a separate set of rats, consisting of a control group (saline injection; *n* = 10 rats) and a CFA group (*n* = 10 rats). Statistical analysis revealed a significant upregulation in the expression of dopamine D1r (*MW* = 10.00, *p* = 0.0190, Fig. 5a) and a downregulation in the expression of the DA DH enzyme (*MW* = 2.00, *p* < 0.0010; Fig. 5d) in the CFA-treated rats compared to the control group. However, no significant differences in mRNA levels were observed between the experimental groups for D2r (*MW* = 21.50, *p* = 0.1455, Fig. 5b)*,* TH (*MW* = 27.00, *p* = 0.1290, Fig. 5c), COMT (*MW* = 15.00, *p* = 0.0760, Fig. 5e), and MAO (*MW* = 48.00, *p* = 0.4559, Fig. 5f).

## Discussion

This study builds upon our previous findings, which demonstrated that inflammatory pain impairs the OFC in rodents, leading to a risk-prone behavior characterized by a preference for uncertain larger outcomes [15, 16]. Here, we examined the relationship between the dopamine D2r and OFC neuronal clusters activity in rats performing the rGT task before and after the onset of prolonged inflammatory pain. The major findings of this study are as follows: (1) The activation or blockade of dopamine D2r of pain-affected animals diminishes their preference for uncertain and large outcomes on the rGT task. (2) This reversal occurs without significant antinociceptive effects, indicating that the behavior changes can be modulated independently from pain relief. (3) These behavior changes were associated with a reorganization of the specialization of OFC neuronal clusters, particularly observed in raclopride-treated rats, resulting in a reduced versatility to encode multiple contingencies. (4) Finally, we also demonstrated that inflammatory pain is associated with alterations in the expression of dopamine D1r and DH enzyme, but not of dopamine D2r, in the OFC. Together, these findings suggest that the disruption of OFC DAergic drive may play a crucial role in regulating the risk-assessment during pain conditions.

The role of the OFC in decision-making processes has been extensively studied in both rodents and humans [4, 8, 12, 25, 43]. Patients with OFC lesions have been reported to exhibit deficient performance in emotional-affective dependent tasks [44], while temporary inactivation of the OFC in rodents has been associated with a selective reduction of irrational choices [45]. Here, we employed a behavioral paradigm called the rGT, which was implemented previously in our laboratory [38]. The rGT is based on the Iowa Gambling task, which is clinically used to investigate gambling-related decision making [46]. Using this paradigm, we have previously demonstrated that rats with bilateral lesions in the OFC or those experiencing inflammatory pain exhibit reduced sensitivity to risk and an increased preference for uncertain and large outcomes, without adequately considering the probability of success in their obtaining [16, 38]. Consistent with these findings, our present data revealed that during the control period of the rGT task, all experimental groups exhibited a clear preference for certain and small outcomes, and this preference was not affected by the administration of D2r ligands. However, after the injection of CFA the rats treated with vehicle shifted their preference to uncertain and larger outcomes. This profile is characterized by an increased basal firing activity of OFC neurons following CFA injection (Fig. 3c), which could, in turn, influence decision-making processes. Human studies have demonstrated that the pain-inhibitory effects of rewards are associated with increased OFC activity [47]. In this context, this mediator function is likely attributable to the integration of information regarding pain value and significance, rather than nociceptive processing, facilitated by interactions between the OFC and other brain areas [25, 48]. Furthermore, recent clinical research has proposed the OFC as a potential neuromarker for chronic pain [49]. Interestingly, both experimental groups treated with D2r ligands maintained and even strengthened their preference for certain and small outcomes without affecting basal firing activity. It is also important to refer that the preference for the risk option observed in the vehicle group after CFA treatment of our study contrasts with the previous findings [15, 16], which reported an incremental preference for the risky option across each testing session. This divergence may be due to differences in task design, training protocols, or the specific impact of inflammatory pain on decision-making strategies, highlighting the need for further investigation.

Previous reports have found that the non-selective D2r antagonist eticlopride improved performance on a rGT paradigm favoring responses to achieve the best profit [50]. In contrast, the D2r agonist quinpirole has been shown to had not significant effect on rGT [50]. The similar behavioral effects of quinpirole and raclopride administrations can be attributed to their shared influence on the U-shaped relationship between DA signaling and behavior [51], where both excessive and insufficient receptor activation lead to comparable disruptions in decision-making and reward processing. Here, we also found that the administration of the D2r ligands at the selected dosage did not affect the number of omissions performed between the control and pain periods. However, it did have a significant impact reducing the response latency for lever press in the rGT task. The absence of motor impairment in our study, despite the observed change in decision-making behavior, can be explained by the specific dosages of the D2r ligands used, which did not interfere with motor functions [42]. This suggests that the changes in behavior were primarily related to motivation, cognitive and reward-processing pathways, rather than to motor function. Another important point to consider is that male rats are naturally more tolerant of scenarios where the higher-risk options offer larger potential outcomes compared to females [52]. However, this inherent risk-prone profile alone does not fully explain the increased preference for high-risk options observed in rats with inflammatory pain compared to control rats without pain.

It has been reported that higher dosages of D2r ligands can significantly affect pain responses or motor activity [17, 42, 53]. However, it is worth noting that the dosage of each D2r ligand used did not result in significant changes in peripheral pain responses or motor activity. The lack of a clear relationship between pain threshold and the dosage of D2r modulators applied suggests that the choice preference profile may not be directly mediated by differences in pain sensivity. Instated, it may be attributed to the reorganization of OFC networks in response to cognitive demand. It has been shown that DA availability in the OFC plays an important role in regulating the exchange and integration of information with other brain regions, which is essential for constantly updating reward value and probability of success during decision making in ambiguous contexts [54–56]. Previous studies have elegantly demonstrated that OFC neurons exhibited remarkable flexibility in adjusting their activity patterns based on the outcome value [57–60]. This process involves the dynamic selective inhibition of critical nodes within the network, facilitating information processing during cognitive tasks [61, 62].

In our study, we observed that the majority of OFC recorded neurons exhibited increased activity during rewarded trials. Moreover, some neurons displayed decreased activity during non-rewarded trials, indicating the encoding of unexpected reward omission. These findings support the role of the OFC in generating reward prediction error signals that regulate the activity of DA neurons [57, 63]. It is well-established that the OFC modulates the firing of DA neurons in the ventral tegmental area (VTA) [55], and reciprocally, the VTA serves as the primary source of DAergic input to the prefrontal areas [64]. To investigate the variability of OFC neuronal responses, we employed an unsupervised hierarchical cluster analysis based on activity signatures. The objective of this analysis was to group neurons with similar response modalities. In contrast to non-rewarded trials, we observed that the majority of the neuronal clusters exhibited increased activity during rewarded trials, with slight variations depending on the ambiguity of the outcome and pharmacological treatment. Moreover, we identified significant differences between the control and pain periods, where OFC clusters displayed reduced flexibility in encoding multiple behavioral responses. This specialization was particularly pronounced in rats treated with raclopride during the pain period. These findings suggest that inflammatory pain may lead to an intra-OFC neuronal reorganization to adapt the representation of outcome value context. Despite the alteration of OFC neuronal integrity, it is important to consider that these functional differences may also be influenced by alterations in other brain areas also important for both reward-related information and pain processing. For instance, the OFC sends excitatory projections to the ventral striatum, which can influence local neural ensembles responsible for encoding outcome value [65]. This modulatory drive exerted over ventral striatum competes with input from other cortical and subcortical areas and may serve as a buffer to control outcome-related information processing [66]. On the other hand, the ventral striatum also shares a strong functional interconnectivity with the OFC [65, 67]. To address whether D2r-related modulation explains differences in functional clusters, future investigations will be needed to examine whether pharmacological D2r manipulation preferentially influences specific neuronal subpopulations within the OFC using also local pharmacological manipulations.

The involvement of the OFC in pain processing makes it unsurprising that abnormalities in its structural phenotype or DAergic transmission could contribute to pain conditions. In our study, we found an upregulation of D1r and a downregulation of DH enzyme, which is responsible for the conversion of DA into norepinephrine. These findings suggest a shift in DAergic signaling that may influence decision-making under pain conditions. Increased D1r expression could enhance reward-driven behaviors through heightened striatal DA activity, while reduced DH levels may limit norepinephrine synthesis, potentially modulating stress and pain responses. Such changes may represent compensatory mechanisms aimed at maintaining DAergic homeostasis during pain. Inadequate availability of DH has been linked to other pathological and neurodegenerative conditions [68, 69], indicating that inflammatory pain may contribute to central neurotransmitter dysfunctions. Additionally, these findings align with the hypothesis that inflammatory pain could suppress neural network activity through enhanced OFC function. This, in turn, may lead to local DA depletion, a hallmark of pathological chronic pain conditions [16, 19], compromising the neural integrity of the OFC [70, 71]. These results underscore the need for future studies to explore the structural and functional specificity of OFC outputs when evaluating the effects of inflammatory pain. Understanding these mechanisms could inform therapeutic strategies targeting pain-induced disruptions in decision-making and reward processing.

In summary, the present study provides new insights into how D2 receptor activity plays an important role in maintaining the complex neural activity balance necessary to sustain OFC network stability and input selectivity for risk-based information processing during cognitive demand. Together, our findings suggest that restoring the D2r-dependent drive of the OFC local network may be a strategy to diminish risk-assessment deficits observed in inflammatory pain conditions.

## Supplementary Information

Below is the link to the electronic supplementary material.

ESM 1 (PDF 1.55 MB)

## Data Availability

No datasets were generated or analysed during the current study.
