# Supplementary material for: Neuromodulation of Dopamine D2 Receptors Alters Orbitofrontal Neuronal Activity and Reduces Risk-Prone Behavior in Male Rats with Inflammatory Pain
Source: Mol Neurobiol. 2025 Feb 22;62(7):8187–203. doi: 10.1007/s12035-025-04781-0 (PMC12209031; doi:10.1007/s12035-025-04781-0)
Supplement: Supplementary file 1 — (PDF 1.55 MB) [file 12035_2025_4781_MOESM1_ESM.pdf]

APPENDIX A – *Supplementary data*

(a)

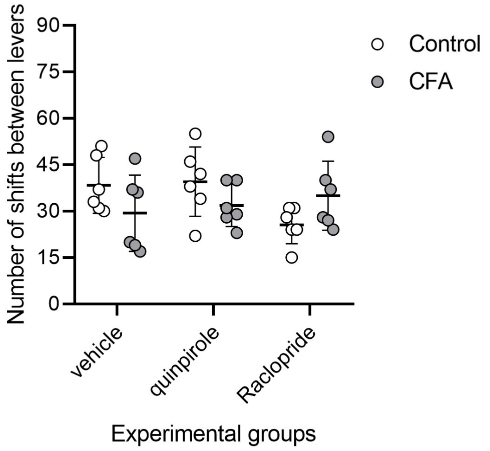

(b)

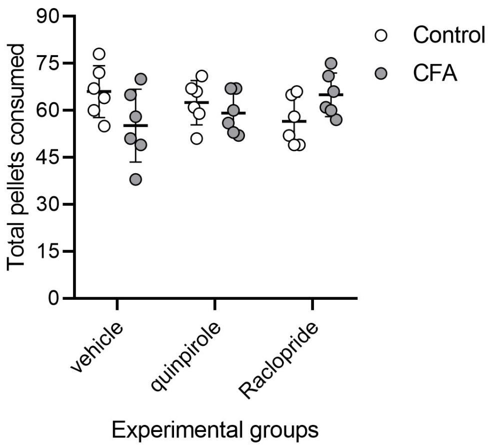

(c)

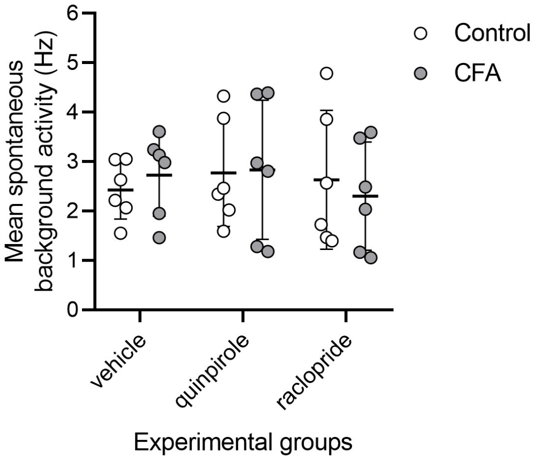

(d)

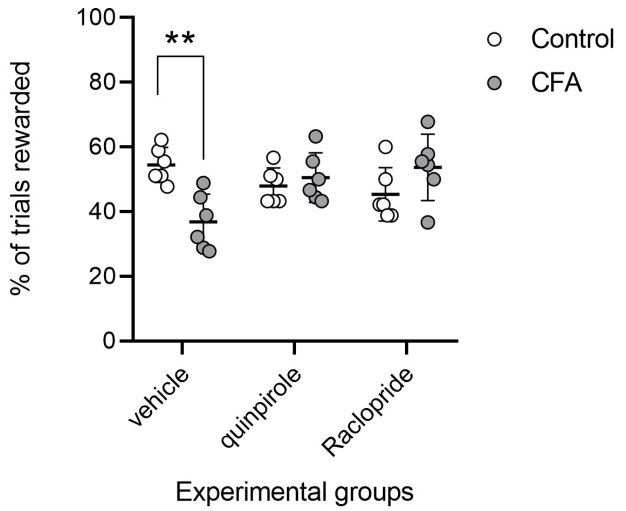

Figure S1 – ***Complementary behavioral data and basal spontaneous OFC activity.*** **(a)** Number of shifts between left and right level. No significant effects were observed between experimental groups or control and CFA rGT sessions (two-way ANOVA – Factor 1: Experimental groups $F_{(2,30)}$=0.54, *p*=0.4668, and Factor 2: control/CFA sessions $F_{(1,30)}$=0.96, *p*=0.3931). **(b)** Total number of pellets consumed per rGT testing session. No significant effects were observed between experimental groups and control and CFA rGT sessions (two-way ANOVA – Factor 1: Experimental groups, $F_{(2,30)}$=0.01, *p*=0.9971, and Factor 2: control/CFA sessions, $F_{(1,30)}$=0.47, *p*=0.4963). **(c)** Mean baseline activity of each rat during a 10-minute open-field arena exposure. No significant differences were observed between experimental groups and control and CFA periods (two-way ANOVA – Factor 1: Experimental groups, $F_{(2,30)}$=0.28, *p*=0.7552, and Factor2: control/CFA sessions, $F_{(1,30)}$=0.01, *p*=0.9750). **(d)** Percentage of rewarded trials per rGT testing session. Analysis of variance showed no significant effects between experimental groups ($F_{(2,30)}$=0.92, *p*=0.4074), and a significant effect between control and CFA rGT testing sessions ($F_{(1,30)}$=9.11, *p*=0.0008); moreover, vehicle-treated rats revealed a decrease of the percentage of rewarded trials following CFA injection (Sidak's *post hoc* test, control versus CFA, p<0.01). Vehicle group *n*=6 rats; Quinpirole group *n*=6 rats, and Raclopride *n*=6 rats. Values are presented as mean ± S.D. ** when *p*<0.01.

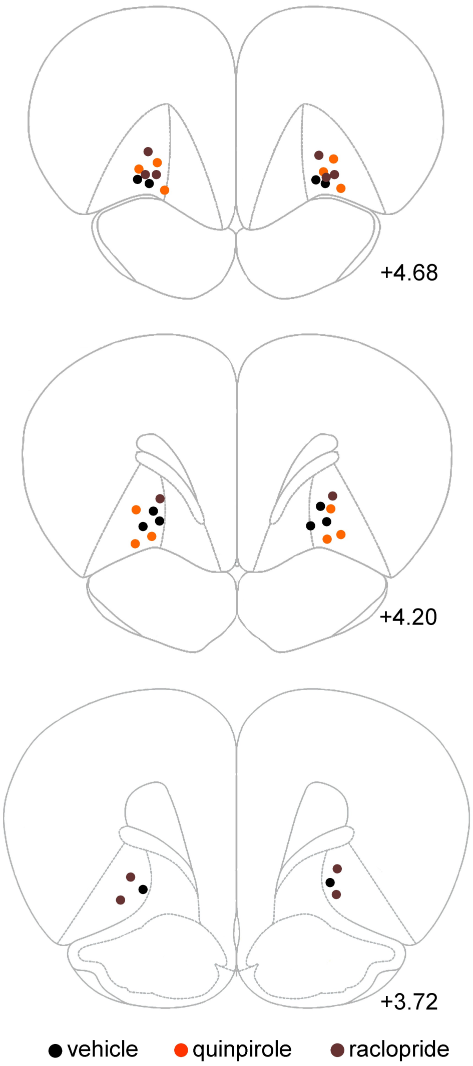

Figure S2 – *Tissue validation of the final location of implanted recording microelectrode arrays.* Coronal diagram illustrating the location of the recording locations in the lateral orbitofrontal cortex (LO). Vehicle group *n*=6, quinpirole group *n*=6, and raclopride group *n*=6. The bottom-right number indicates the anterior distance (in millimeters) relative to bregma.

Table S1 – *Classification of recorded units based on their spontaneous firing pattern and waveform characteristics.*

Experimental Group		Total single-units recorded (*n*)	Excitatory neurons (*n* / %)	Mean FR±SEM	Inhibitory neurons (n / %)	Mean FR±SEM	Unclassified (n / %)
Vehicle	Control	81	74 / 91.35%	1.66±0.48	4 / 4.94%	5.11±1.82	3 / 3.71%
	CFA		73 / 90.13%	2.95±1.46	6 / 7.40%	6.71±2.33	2 / 2.47%
Quinpirole	Control	80	71 / 88.75%	2.56±1.08	5 / 6.25%	5.91±2.05	4 / 5.00%
	CFA		72 / 90.00%	2.74±1.36	6 / 7.50%	6.03±0.84	2 / 2.50%
Raclopride	Control	78	69 / 88.47%	2.89±1.14	7 / 8.97%	5.48±1.68	2 / 2.56%
	CFA		71 / 91.02%	3.02±1.02	4 / 5.13%	6.58±2.62	3 / 3.85%

Recorded units were classified as excitatory neurons if they displayed a spike width (peak-to-through) > 450 µs, signal-to-noise ratio > 3:1, and a mean firing rate < 5 Hz; and classified as inhibitory neurons if they displayed a spike width > 250 µs, signal-to-noise ratio > 2:1, and a mean firing rate > 5 Hz. Units that did not match either of these criteria were labeled -unclassified-.
